# Vertical distribution of arthropod assemblages in native and exotic forests of Terceira Island (Azores, Portugal)

**DOI:** 10.3897/BDJ.13.e154240

**Published:** 2025-05-20

**Authors:** Sébastien Lhoumeau, Abrão Leite, Laurine Parmentier, Clémence Massard, Martha Vounatsi, Georgery Lucie, Paulo A. V. Borges

**Affiliations:** 1 University of the Azores, cE3c- Centre for Ecology, Evolution and Environmental Changes/Azorean Biodiversity Group, CHANGE – Global Change and Sustainability Institute, School of Agricultural and Environmental Sciences, Rua Capitão João d'Ávila, Pico da Urze, 9700-042, Angra do Heroísmo, Azores, Portugal University of the Azores, cE3c- Centre for Ecology, Evolution and Environmental Changes/Azorean Biodiversity Group, CHANGE – Global Change and Sustainability Institute, School of Agricultural and Environmental Sciences, Rua Capitão João d'Ávila, Pico da Urze, 9700-042 Angra do Heroísmo, Azores Portugal; 2 Rua Fernando Pessoa, nº99 R/C DTO 2765-483, Estoril, Portugal Rua Fernando Pessoa, nº99 R/C DTO 2765-483 Estoril Portugal; 3 Mestrado em Gestão e Conservação da Natureza, University of the Azores Rua Capitão João d'Ávila, Pico da Urze 9700-042, Angra do Heroísmo, Azores, Portugal Mestrado em Gestão e Conservação da Natureza, University of the Azores Rua Capitão João d'Ávila, Pico da Urze 9700-042 Angra do Heroísmo, Azores Portugal; 4 Department of Ecology and Taxonomy, Faculty of Biology, National and Kapodistrian University of Athens, Athens, Greece Department of Ecology and Taxonomy, Faculty of Biology, National and Kapodistrian University of Athens Athens Greece; 5 UCLouvain - Unamur, Faculty of Biology, Louvain-La-Neuve, Belgium UCLouvain - Unamur, Faculty of Biology Louvain-La-Neuve Belgium; 6 IUCN SSC Atlantic Islands Invertebrate Specialist Group, Angra do Heroísmo, Azores, Portugal IUCN SSC Atlantic Islands Invertebrate Specialist Group Angra do Heroísmo, Azores Portugal; 7 IUCN SSC Monitoring Specialist Group, Angra do Heroísmo, Azores, Portugal IUCN SSC Monitoring Specialist Group Angra do Heroísmo, Azores Portugal

**Keywords:** occurrence, specimen, Arthropoda, Azores, forest stratification, SLAM trap, Pitfall trap, sampling event

## Abstract

**Background:**

In the summer of 2024, a study was conducted on Terceira Island in the Azores Archipelago, Portugal, aiming to characterise the vertical diversity and spatial distribution patterns of arthropods within native and exotic forest ecosystems. This study forms part of a broader research initiative designed to investigate how alterations in habitat structure influence the complexity and stability of arthropod food webs in Azorean forest habitats. By systematically sampling arthropods across multiple vertical strata —from forest floor to canopy the study aimed to generate detailed insights into the ecological dynamics governing biodiversity patterns and species interactions. Results from this monitoring will contribute significantly to understanding the ecological impacts of forest composition and management strategies, ultimately providing information for conservation planning and habitat restoration efforts aimed at preserving arthropod diversity and ecological resilience in island ecosystems.

**New information:**

The current dataset comprises identified terrestrial arthropods collected using SLAM (Sea, Land and Air Malaise) traps and Pitfall traps across diverse forest strata. A total of 32,797 specimens were collected from the Arachnida, Diplopoda, Chilopoda and Insecta classes. A total of 18,372 (56%) were identified at the species or subspecies level, including 12,745 adults and 5,627 juveniles for taxa, such as Araneae and Hemiptera due to the availability of reliable identification methods. The resulting dataset encompasses 150 species and 11 subspecies, distributed across 21 orders, 81 families and 148 genera.

Hemiptera emerged as the most abundant identified order, with a total of 7,697 recorded specimens and Coleoptera stood as the most taxonomically diverse, encompassing 19 distinct families and 50 species and sub-species. The ten most abundant species comprise predominantly endemic and native non-endemic species, with two exotic species detected amongst them.

This comprehensive dataset serves as a significant augmentation of the existing baseline knowledge concerning the diversity of Azorean arthropods, thereby facilitating the formulation of future long-term ecological comparisons. It offers valuable insights into the vertical distribution of species abundance within both native and exotic forests of the Azores.

## Introduction

Forests represent amongst the most structurally complex ecosystems on Earth (Pan et al. 2013, Ehbrecht et al. 2021), characterised by distinct vertical strata that support a wide range of biodiversity (Oliveira and Scheffers 2019). The vertical stratification of forests plays a crucial role in shaping species distributions, ecological interactions and resource availability (Laurans et al. 2014, Thiel et al. 2021, Basham et al. 2023). The different forest layers — ranging from the forest floor to the canopy — offer distinct environmental conditions, including variations in temperature, humidity, light availability and plant composition (Chen et al. 1999, De Frenne et al. 2019). Consequently, many forest-dwelling organisms, including arthropods, exhibit strong vertical preferences and niche partitioning (Basset et al. 2003). However, despite the recognised importance of vertical stratification in forest ecology, studies on arthropod diversity across forest layers remain limited, especially in insular ecosystems (but see Costa et al. (2023)).

Arthropods are considered to be one of the most functionally diverse and ecologically significant animal groups. They play key roles in decomposition, pollination, herbivory, predation and soil aeration (Wong et al. 2019, Cardoso et al. 2020, Cardoso et al. 2024). Due to their sensitivity to habitat structure and environmental changes, arthropods are widely used as bioindicators of ecosystem health (Tsafack et al. 2023). Understanding their distribution across vertical forest layers can provide insights into species interactions, habitat specialisation and the effects of environmental disturbances on biodiversity. In island ecosystems, where species assemblages are often shaped by historical colonisation events, habitat fragmentation and the introducion of invasive species (Fernández-Palacios et al. 2021), investigating arthropod vertical stratification can be particularly valuable for conservation planning. Island ecosystems, such as those of the Azores (Portugal), exhibit unique biodiversity patterns, shaped by isolation, habitat heterogeneity and anthropogenic influences, making them valuable natural laboratories for ecological reserach and biodiversity management strategies (Mueller-Dombois 1992).

The Azorean forests, include both native and exotic forest types, each of which differs in terms of floristic composition, structural complexity and historical land use (Dias et al. 2004, Elias et al. 2016, Borges Silva et al. 2022). The native forests, which are dominated by endemic tree species, such as *Laurusazorica*, *Ilexazorica* and *Juniperusbrevifolia*, represent remnants of the Pliocene/Pleistocene forests in Macaronesia (Kondraskov et al. 2015). These forests are distinguished by their notable levels of endemism and conservation importance, offering critical habitat for specialised arthropod species (Borges et al. 2022, Lhoumeau and Borges 2023). In contrast, exotic forests are characterised by the presence of the invasive species *Pittosporumundulatum*, along with other non-native vascular plants and are the result of deliberate afforestation for timber production and land management purposes (Dias et al. 2004, Borges Silva et al. 2017). As a consequence, they frequently exhibit a lack of structural and botanical diversity when compared to native forests, potentially influencing the composition and distribution of arthropod communities.

Given that many insular arthropods exhibit high levels of habitat specialisation and restricted dispersal abilities (Gillespie and Roderick 2002), their vertical distribution within forest strata could be influenced by both natural forest structure and anthropogenic modifications. Additionally, the replacement of native forests with exotic species may lead to changes in arthropod assemblages by altering microhabitat conditions, reducing resource availability and disrupting ecological interactions.

## General description

### Purpose

The present dataset encompasses terrestrial arthropods that have been collected using Pitfall traps and SLAM (Sea, Land, and Air Malaise) traps across a variety of forest strata. This dataset is the material result of sampling events that have been conducted within the framework of a project that aims to evaluate the impact of habitat structure change on arthropod food web complexity in Azorean forests. In particular, the study seeks to assess how changes in arthropod biodiversity are influenced by the structural complexity of forests.

## Project description

### Title

The impact of habitat structure change on arthropod food web complexity in Azorean forests.

### Personnel

Paulo A. V. Borges, Sébastien Lhoumeau, Laurine Parmentier, Abrão Leite, Clémence Massard, Martha Vounatsi, Georgery Lucie

The project was conceived and is being led by Sébastien Lhoumeau and Paulo A.V. Borges.

Fieldwork (site selection and experimental setting): Sébastien Lhoumeau and Paulo A.V. Borges.

Fieldwork (authorisation): Licença Nº 23/2024/DRAAC; ADENDA CCIR-RAA/2024/7.

Fieldwork: Sébastien Lhoumeau, Clémence Massard, Martha Vounatsi, Georgery Lucie and Paulo A.V. Borges.

Parataxonomists (Laboratory): Sébastien Lhoumeau, Laurine Parmentier, Abrão Leite, Clémence Massard, Martha Vounatsi, Georgery Lucie.

Taxonomists: Paulo A. V. Borges.

Arthropod Curation: Voucher specimen management was mainly undertaken by Sébastien Lhoumeau, Laurine Parmentier and Abrão Leite.

Darwin Core Databases: Sébastien Lhoumeau and Paulo A.V. Borges.

### Study area description

The Azores constitute an isolated archipelago located in the northern part of the mid-Atlantic Ocean, approximately 1,400 kilometres west of mainland Portugal. Comprising nine volcanic islands — namely Corvo, Flores, Faial, Pico, São Jorge, Graciosa, Terceira, São Miguel and Santa Maria — the Archipelago extends across roughly 500 km in a west-northwest to east-southeast orientation. Santa Maria, with its age around 6 to 8 million years, is the most ancient island within the archipelago. In contrast, Pico, the youngest island, has an estimated age of around 0.19 million years (Florencio et al. 2021). The islands emerged through volcanic activity along the Mid-Atlantic Ridge, a tectonic boundary zone, characterised by ongoing seismic and geothermal phenomena and most of the islands are relatively young (Florencio et al. 2021). This volcanic origin has endowed the Azorean Islands with rugged terrains, diverse habitats and unique ecological communities, which together contribute to their important uinque biodiversity and biogeographic significance (Florencio et al. 2021, Borges et al. 2022).

During this project, the Island of Terceira (the third largest) was surveyed. Ten sampling plots were selected in areas of native vegetation, predominantly dominated by endemic species such as *Juniperusbrevifolia*, *Ericaazorica*, *Laurusazorica* and *Ilexazorica*, with currently some spread of invasive species like *Hedychiumgardnerianum*. Ten additional plots were situated in secondary forests, predominantly characterised by *Pittosporumundulatum* and *Hedychiumgardnerianum*, yet exhibiting indications of endemic and native ferns, such as *Dryopterisazorica* and *Diplaziumcaudatum*.

### Design description

The experimental design comprised a 90-day sampling period, spanning from mid-June to mid-September 2024 (summer period), across all twenty sites. The sampling method employed was SLAM traps, with a maximum of three traps deployed at each site. In locations where feasible, these traps were positioned at varying heights within the forest, specifically at 0% (ground trap, hereafter GRD), 50% (understorey trap, UND) and 75% (canopy trap, CAN) of the maximum canopy height. In the event that the understorey trap was separated from the other two traps by less than 1 vertical metre, this trap was not set up.

Additionally, 14 Pitfall traps (hereafter EPI) were randomly set up at each site for a duration of 14 days, starting in July and concluding in August 2024.

### Funding

Sebastien Lhoumeau was funded by the project ”The impact of habitat structure change on arthropod food web complexity in Azorean forests” (PhD grant M3.1.a/F/012/2022).

Additional funding come for :

Portal da Biodiversidade dos Açores (2022-2023) - PO Azores Project - M1.1.A/INFRAEST CIENT/001/2022;

FCT-UIDB/00329/2020-2024 (Thematic Line 1 – integrated ecological assessment of environmental change on biodiversity) (2019-2024);

Science and Technology Foundation (FCT) - MACRISK-Trait-based prediction of extinction risk and invasiveness for Northern Macaronesian arthropods (FCT-PTDC/BIA-CBI/0625/2021).

Open access was funded by the project FCT-UID/00329/2025, Centre for Ecology, Evolution and Environmental Changes (CE3C).

## Sampling methods

### Study extent

A total of twenty 20 m x 20 m plots were sampled in one island from the Archipelago (Terceira). Ten of these plots were set up within the most well-preserved forests in this island, having limited human disturbance (Borges et al. 2017). The native forest is dominated by endemic vegetation, such as *Juniperusbrevifolia*, *Ericaazorica*, *Laurusazorica* and *Ilexazorica* (see Borges et al. (2017) for more details). Ten other plots are in secondary forests, which are dominated by exotic and invasive trees.

### Sampling description

**Figure 1.**
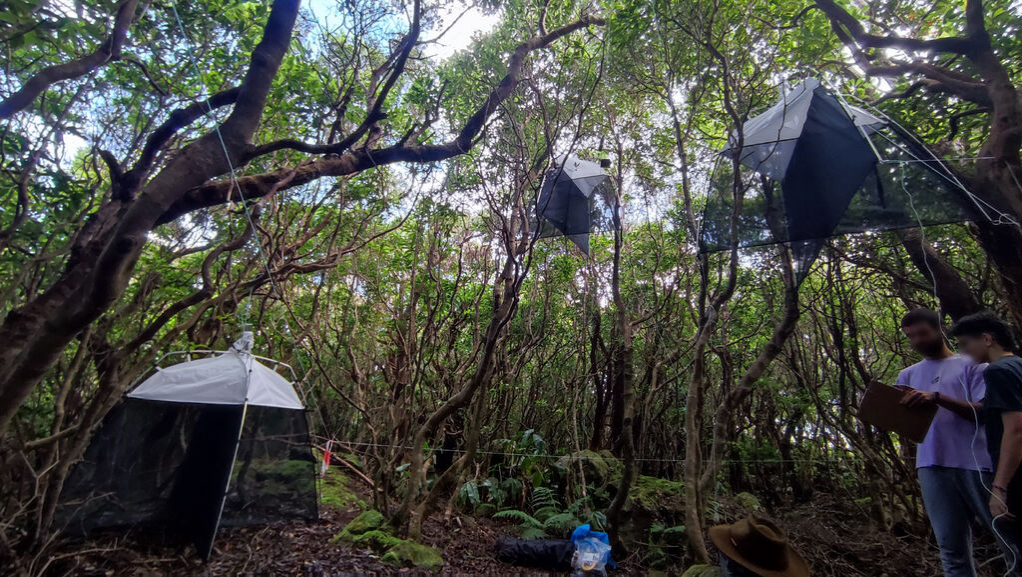
Picture of the set-up of the three SLAM traps within the exotic forest (site TER-EXO-T04) (Credit: Sébastien Lhoumeau).

Passive flight interception SLAM traps (Sea, Land and Air Malaise trap, Fig. 1) were used to sample the plots, with three traps being set up at each plot at different height within the forest. Traps are 110 × 110 × 110 cm. In this type of trap, the trapped arthropods crawl up the mesh and then fall inside the sampling recipient (Borges et al. 2017). Each one is filled with propylene glycol (pure 1,2-PROPANODIOL) to kill the captured arthropods and conserve the sample between collections, enabling also the preservation of DNA for future genetic analysis. Although this protocol was developed to sample flying arthropods, by working as an extension of the tree, non-flying species, such as spiders, can also crawl into the trap (Borges et al. 2017), enhancing the range of groups that can be sampled by this technique. As a result, previous studies have used these traps to analyse diversity and abundance changes in the arthropod communities in Azores pristine forest sites (Matthews et al. 2019, Borges et al. 2020, Lhoumeau and Borges 2023). The traps samples were collected after three months in the studied sites.

**Figure 2.**
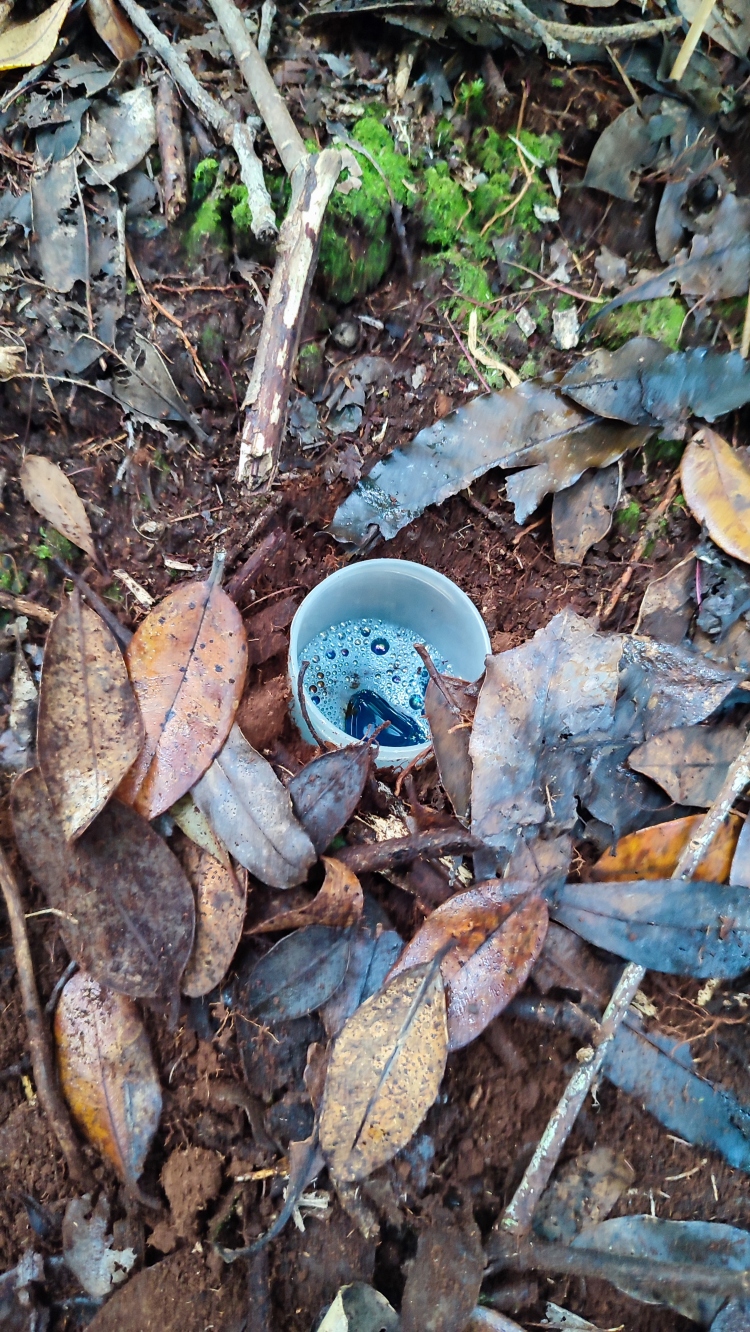
Picture of a Pitfall trap set-up (the protective cover is removed) (Credit: Sébastien Lhoumeau).

We completed the sampling by using 14 passive Pitfall traps (Fig. 2) randomly distributed within the plots to sample the epigean fauna. Traps have a 5 cm opening diameter and filled with ethylene glycol. Pitfall traps were collected after two weeks (14 nights) of continuous operation.

### Quality control

All sorted specimens were identified by a taxonomical expert, one of the authors P.A.V.B. and species taxonomic nomenclature and species colonisation status follows Borges et al. (2022).

## Geographic coverage

### Description

**Figure 3.**
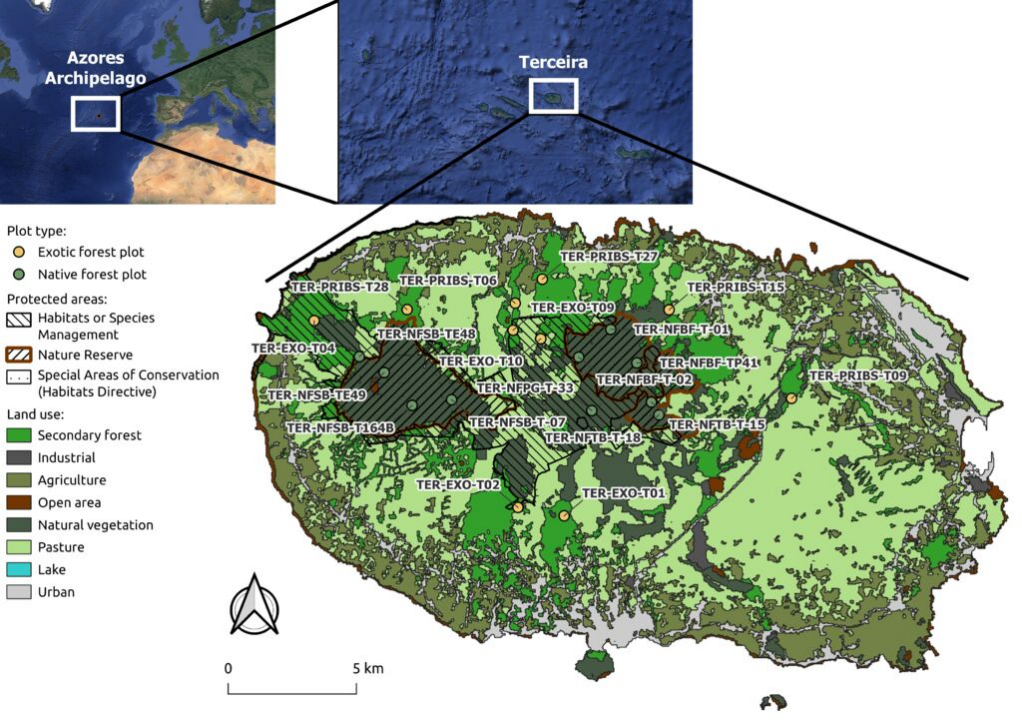
Location of Terceira Island. For comprehensive details regarding the sampling sites, refer to Table 1. The protected areas data was sourced from UNEP-WCMC (2025), while the land-use data were provided by the Azorean government.

Terceira Island, Azores (Portugal), Fig. 3.

### Coordinates

-27.04093 and -27.39698 Latitude; 38.81982 and 38.62170 Longitude.

## Taxonomic coverage

### Description

The following orders and class are covered:

### Taxa included

**Table:** 

Rank	Scientific Name	
kingdom	Animalia	
phylum	Arthropoda	
class	Insecta	
class	Arachnida	
class	Diplopoda	
class	Chilopoda	
order	Coleoptera	
order	Hemiptera	
order	Psocodea	
order	Araneae	
order	Neuroptera	
order	Hymenoptera	
order	Thysanoptera	
order	Archaeognatha	
order	Opiliones	
order	Pseudoscorpiones	
order	Phasmida	
order	Dermaptera	
order	Julida	
order	Blattodea	
order	Lepidoptera	
order	Ephemeroptera	
order	Trichoptera	
order	Lithobiomorpha	
order	Geophilomorpha	
order	Polydesmida	
order	Strepsiptera	

## Temporal coverage

**Data range:** 2024-6-11 – 2024-9-27.

### Notes

SLAM traps were collected after three months in the studied sites. Pitfall traps were recovered after two weeks (14 nights) of continuous operation.

## Collection data

### Collection name

Entomoteca Dalberto Teixeira Pombo

### Collection identifier

DTP

### Specimen preservation method

Ethanol (96%)

## Usage licence

### Usage licence

Creative Commons Public Domain Waiver (CC-Zero)

## Data resources

### Data package title

Stratified sampling of Azorean forest arthropods

### Resource link

https://doi.org/10.15468/7aue4t

### Alternative identifiers

https://www.gbif.org/dataset/d3580ac2-a504-44e0-8e59-de89c430924c; http://ipt.gbif.pt/ipt/resource?r=azores_forest_arthropods

### Number of data sets

2

### Data set 1.

#### Data set name

Event table

#### Data format

Darwin Core Archive format

#### Character set

UTF-8

#### Download URL

http://ipt.gbif.pt/ipt/archive.do?r=azores_forest_arthropods

#### Data format version

Version 1.6

#### Description

The dataset was published in the Global Biodiversity Information Facility platform, GBIF (Lhoumeau and Borges 2025). The following data-table includes all the records for which a taxonomic identification of the species was possible. The dataset submitted to GBIF is structured as a sample event dataset that has been published as a Darwin Core Archive (DwCA), which is a standardised format for sharing biodiversity data as a set of one or more data tables. The core data file contains 326 records (eventID). This GBIF IPT (Integrated Publishing Toolkit, Version 2.5.6) archives the data and, thus, serves as the data repository. The data and resource metadata are available for download in the Portuguese GBIF Portal IPT (Lhoumeau and Borges 2025).

**Data set 1.** 

Column label	Column description
id	Unique identification code for sampling event data.
eventID	Identifier of the events, unique for the dataset.
samplingProtocol	The sampling protocol used to capture the species.
sampleSizeValue	The numeric amount of time spent in each sampling.
sampleSizeUnit	The unit of the sample size value.
eventDate	Date or date range the record was collected.
eventRemarks	The verbatim original representation of the date and time information for an Event. In this case, we use the season and year.
habitat	The habitat from which the sample was obtained.
locationID	Identifier of the location.
islandGroup	Name of archipelago, always Azores in the dataset.
island	Name of the island, always Terceira in the dataset.
country	Country of the sampling site, always Portugal in the dataset.
countryCode	ISO code of the country of the sampling site, always PT in the dataset.
stateProvince	Name of the region of the sampling site.
municipality	Municipality of the sampling site.
locality	Name of the locality.
minimumElevationInMetres	The lower limit of the range of elevation (altitude, above sea level), in metres.
locationRemarks	Details on the locality site.
decimalLatitude	Approximate decimal latitude of the trap.
decimalLongitude	Approximate decimal longitude of the trap.
geodeticDatum	The ellipsoid, geodetic datum or spatial reference system (SRS) upon which the geographic coordinates given in decimalLatitude and decimalLongitude are based, always WGS84 in the dataset.
coordinateUncertaintyInMetres	Uncertainty of the coordinates of the centre of the sampling plot.
coordinatePrecision	Precision of the coordinates.
georeferenceSources	A list (concatenated and separated) of maps, gazetteers or other resources used to georeference the Location, described specifically enough to allow anyone in the future to use the same resources.

### Data set 2.

#### Data set name

Occurrence table

#### Data format

Darwin Core Archive format

#### Character set

UTF-8

#### Download URL

http://ipt.gbif.pt/ipt/resource?r=azores_forest_arthropods

#### Data format version

Version 1.6

#### Description

The dataset was published in the Global Biodiversity Information Facility platform, GBIF (Lhoumeau and Borges 2025). The following data table includes all the records for which a taxonomic identification of the species was possible. The dataset submitted to GBIF is structured as an occurrence table that has been published as a Darwin Core Archive (DwCA), which is a standardised format for sharing biodiversity data as a set of one or more data tables. The core data file contains 2399 records (occurrenceID). This GBIF IPT (Integrated Publishing Toolkit, Version 2.5.6) archives the data and, thus, serves as the data repository. The data and resource metadata are available for download in the Portuguese GBIF Portal IPT (Lhoumeau and Borges 2025).

**Data set 2.** 

Column label	Column description
id	Unique identification code for species abundance data. Equivalent here to eventID.
type	The nature or genre of the resource, as defined by the Dublin Core standard. In our case "PhysicalObject".
licence	Reference to the licence under which the record is published.
institutionID	The identity of the institution publishing the data.
collectionID	The identity of the collection where the specimen are conserved.
collectionID	The identity of the collection publishing the data.
institutionCode	The code of the institution publishing the data.
collectionCode	The code of the collection where the specimens are conserved.
datasetName	Name of the dataset.
basisOfRecord	The nature of the data record.
recordedBy	A list (concatenated and separated) of names of peoples, groups or organisations who performed the sampling in the field.
occurrenceID	Identifier of the record, coded as a global unique identifier.
organismQuantity	A number or enumeration value for the quantity of organisms.
organismQuantityType	The type of quantification system used for the quantity of organisms.
sex	The sex and quantity of the individuals captured.
lifeStage	The life stage of the organisms captured.
establishmentMeans	The process of establishment of the species in the location, using a controlled vocabulary: 'native', 'introduced', 'endemic' or 'indeterminate'.
eventID	Identifier of the events, unique for the dataset.
identifiedBy	A list (concatenated and separated) of names of people, groups or organisations who assigned the taxon to the record.
dateIdentified	The date on which the subject was determined as representing the taxon.
scientificName	Complete scientific name including author and year.
kingdom	Kingdom name.
phylum	Phylum name.
class	Class name.
order	Order name.
family	Family name.
genus	Genus name.
specificEpithet	Specific epithet
infraspecificEpithet	Infraspecific epithet.
taxonRank	Lowest taxonomic rank of the record.
scientificNameAuthorship	Name of the author of the lowest taxon rank included in the record.
identificationRemarks	Information about morphospecies identification (code in Dalberto Teixeira Pombo Collection).

## Additional information

We collected a total of 32,797 specimens of terrestrial arthropods using SLAM and Pitfall traps deployed across diverse forest strata in native and exotic forests. These specimens, representing the classes Arachnida, Diplopoda, Chilopoda and Insecta, provide a comprehensive snapshot of Azorean arthropod diversity. Of the total collected, 18,372 individuals (56%) were identified at the species or subspecies level — comprising 12,745 adults and 5,627 juveniles (Table 2).

In general, the most abundant order identified was Hemiptera, with 21,939 recorded specimens, underscoring its prevalence in these forest ecosystems. Although not the most abundant, Coleoptera emerged as the most taxonomically diverse group, being represented by 19 distinct families and 50 species and sub-species. The ten most abundant species are predominantly endemic and native non-endemic taxa, with only two introduced species amongst them. This comprehensive dataset significantly augments the existing baseline knowledge on Azorean arthropods and offers valuable insights into the vertical distribution of species abundance within both native and exotic forests.

**Figure 4.**
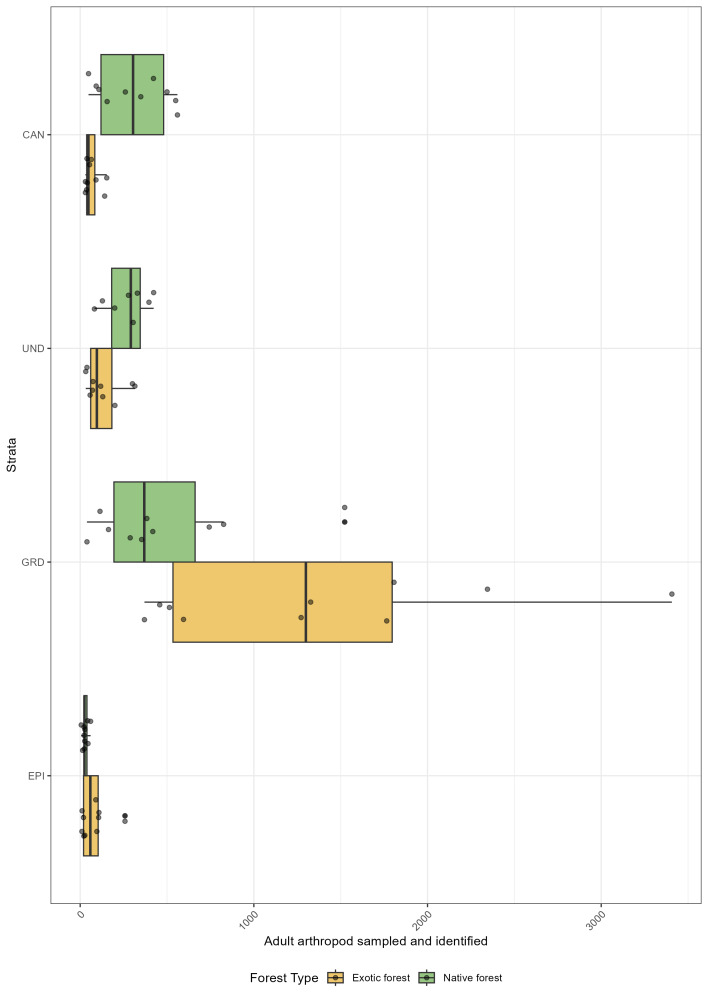
Abundance of arthropods across different forest strata in exotic and native forests. The x-axis represents the total number of arthropods collected and identified, while the y-axis indicates the sampled strata (EPI: epigean, GRD: ground, UND: understorey, CAN: canopy). Points represent individual site values for a given forest type. Bars are colour-coded to distinguish between exotic forests (yellow) and native forests (green). Kruskal-Wallis tests revealed statistically significant differences in adult abundance total across the strata of exotic forest (χ2(3) = 24.2, n = 40, p < 0.001) and native forest (χ2(3) = 20.8, n = 38, p < 0.001). In exotic forest, Kruskal-Wallis effect size (η2[H]) for the difference in adult abundance total was 0.59 (95% CI [0.35, 0.80], n = 40), indicating a large effect. In native forest, the effect was also large with 0.52 (95% CI [0.29, 0.73]).

The dataset provides strong evidence that arthropod communities are structured differently along the vertical gradient in native and exotic forests (Fig. 4). When comparing these two types of forests, we found that distribution of arthropod abundance vary significantly across the three forest strata: ground, understorey and canopy (Table 3).

One of the most striking findings is the more even distribution of arthropods across the vertical strata in native forests compared to exotic forests, where abundance is disproportionately concentrated in the ground layer (Fig. 4).

**Figure 5.**
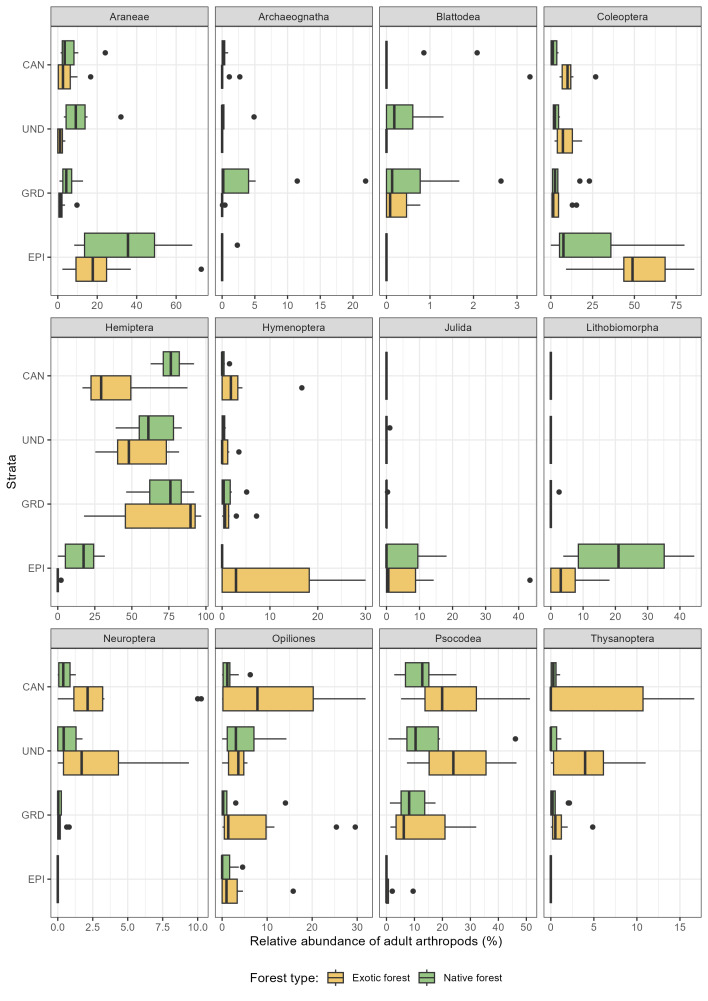
Arthropod vertical profiles in exotic and native forests for the 12 most abundant order sampled and identified. Each panel represents the distribution of the relative abundance (%) of a given order to the total number of individuals sampled in a forest strata (EPI: epigean, GRD: ground, UND: understorey, CAN: canopy) of exotic forests (yellow) and native forests (green).

Native forests offer a greater number of distinct ecological niches at varying heights, thus allowing for a greater degree of vertical partitioning amongst arthropod communities (Basset et al. 2003, Basset et al. 2015, Xing et al. 2023). Therefore, it was hypothesised that the distribution of the overall arthropod assemblage would differ more from one strata to another. However, the observed homogeneity in the abundance distribution could be attributed to the relatively low canopy height within the study plots (Dias et al. 2004, Elias et al. 2016). The well-developed understorey and dense canopy create a structurally complex environment that supports a high diversity of arthropods. The presence of climbing vegetation, epiphytes and diverse leaf architecture contributes to habitat complexity and homogeneity by providing multiple pathway for species to move in the ecosystems. However, when distinguishing the overall arthropod assemblage by order, we detected that native forests are supporting a higher proportion of canopy- and understorey-associated taxa (Fig. 5). Similarly to a study conducted in Amazonian forest by de Souza Amorim et al. (2022), groups such as Araneae, Hemiptera and Hymenoptera (formicidae) show significantly higher relative abundance in the upper strata (Table 4), suggesting that these layers serve as critical habitat for these functional groups (see Arvidsson et al. (2022) on spider's web). The increased presence of predators (e.g. spiders) in the canopy and understorey of native forests may indicate a more complex food web structure, with stronger top-down regulation of herbivore populations (Martinez‐Almoyna et al. 2024, Wildermuth et al. 2024).

In the Azores, exotic forests are dominated by fast-growing, homogeneous tree species and lack the complex understorey and dense canopy of native forests (Connor et al. 2012, Borges Silva et al. 2017, Borges Silva et al. 2018). It is hypothesised that the combination of elevated canopy height and an absence of vertically structural elements may lead to a heightened degree of microclimatic differentiation, which, in turn, may result in a more pronounced vertical stratification within arthropod communities. Additionally, arthropod abundance is significantly higher in the ground layer of exotic forests compared to native forests (Table 3), suggesting that these simplified forest structures concentrate arthropod activity near the forest floor. Many arthropod orders, including detritivores (Julida, Lithobiomorpha) and scavengers (Psocodea), concentrated in the ground layer (Fig. 5). The significantly higher abundance of these groups in the lower strata (Table 4) suggests that exotic forests may be more reliant on decomposition-based energy pathways rather than complex trophic interactions involving arboreal predators and herbivores. This shift could have important implications for ecosystem functioning, potentially leading to altered nutrient cycling and reduced ecological resilience. In addition, the number of specimens sampled from the ground layer in exotic forests might be link to the invasion pattern previously documented by Cardoso et al. (2007), where most of the species appeared to be non-indigenous in this ecosystem.

Overall, our study present, for the first time, a comprehensive stratified survey of forest arthropods in two different forest ecosystem in the Azores Archipelago. The significant differences observed in both overall abundance and order-level composition across strata provide strong evidence that these two forest type are not ecologically equivalent highlighting the need to preserve native forests and enhance vertical complexity in exotic forest to sustain arthropod biodiversity and ecosystem services in forested landscapes. Additionally, future studies should assess how forest structure, microclimatic conditions and resource availability shape arthropod vertical distribution.

## Figures and Tables

**Table 1.** List of the 20 sampled sites in Terceira. Information about the habitat, location identifier, locality, decimal coordinates and elevation in metres are provided. In the habitat, we classify the type of native forest based on Elias et al. (2016): (1) *Laurus* Submontane Forests, (2) *Juniperus*-*Ilex* Montane Forests, (3) *Juniperus* Montane Woodlands. Exotic forests are dominated by the invasive tree species *Pittosporumundulatum*. Elevation data are sourced from OpenTopography (2013). In locations indicated by the asterisk, only two SLAM traps were installed. The implementation of the understorey trap was rendered unfeasible due to the reduced canopy height.

Habitat type	Site code	Site name	Decimal longitude	Decimal latitude	Elevation above sea level (m)
Exotic forest	TER-EXO-T01	Mata do Estado	-27.24	38.697	425
Exotic forest	TER-EXO-T02	Matela	-27.26	38.7	394
Exotic forest	TER-EXO-T04	Serreta 400	-27.352	38.765	376
Exotic forest	TER-EXO-T09	Caparica Horses	-27.263	38.762	417
Exotic forest	TER-EXO-T10	Gruta do Balcões	-27.25	38.759	459
Exotic forest	TER-PRIBS-T06	Caparica	-27.262	38.771	336
Exotic forest	TER-PRIBS-T09	Fontinhas	-27.138	38.738	256
Exotic forest	TER-PRIBS-T15	Agualva	-27.193	38.769	367
Exotic forest	TER-PRIBS-T27	Gruta Chocolade	-27.249	38.779	271
Exotic forest	TER-PRIBS-T28	Pico Rachado	-27.31	38.769	461
Native forest (1)	TER-NFBF-T-01	Morro Assombrado	-27.219	38.762	680
Native forest (3)	TER-NFBF-T-02 (*)	Biscoito da Ferraria	-27.233	38.752	590
Native forest (3)	TER-NFBF-TP41	Pico Alto	-27.207	38.75	673
Native forest (2)	TER-NFPG-T-33	Pico Galhardo	-27.227	38.734	643
Native forest (2)	TER-NFSB-T-07	Lomba	-27.29	38.737	683
Native forest (3)	TER-NFSB-T164B	Santa Bárbara	-27.308	38.735	899
Native forest (3)	TER-NFSB-TE48	Lagoinha	-27.331	38.752	678
Native forest (3)	TER-NFSB-TE49 (*)	Lagoa do Pinheiro	-27.331	38.752	927
Native forest (1)	TER-NFTB-T-15	Terra Brava A	-27.201	38.736	637
Native forest (1)	TER-NFTB-T-18	Terra Brava B	-27.197	38.732	679

**Table 2.** Number of individuals sampled and identified at the species or subspecies level. CAN: canopy layer, UND: understorey layer, GRD: ground layer, EPI: epigean layer. Epigean layer is sampled with pitfall traps whereas all the other layers are sampled with SLAM traps. Establishment (species colonisation status) data is according to Borges et al. (2022).

Class	Order	Scientific Name	Establishment	EPI	GRD	UND	CAN
Arachnida	Araneae	*Acorigoneacoreensis* (Wunderlich, 1992)	endemic	2	17	6	5
Arachnida	Araneae	*Agalenatearedii* (Scopoli, 1763)	introduced	0	2	0	0
Arachnida	Araneae	*Agynetadecora* (O. Pickard-Cambridge, 1871)	introduced	2	1	0	0
Arachnida	Araneae	*Canariphantesacoreensis* (Wunderlich, 1992)	endemic	207	7	0	0
Arachnida	Araneae	*Cheiracanthiumerraticum* (Walckenaer, 1802)	introduced	0	3	1	2
Arachnida	Araneae	*Clubionaterrestris* Westring, 1851	introduced	0	8	0	0
Arachnida	Araneae	*Cryptachaeablattea* (Urquhart, 1886)	introduced	0	11	2	0
Arachnida	Araneae	*Dysderacrocata* C. L. Koch, 1838	introduced	119	18	2	0
Arachnida	Araneae	*Erigoneatra* Blackwall, 1833	introduced	1	0	0	0
Arachnida	Araneae	*Erigonedentipalpis* (Wider, 1834)	introduced	0	1	0	0
Arachnida	Araneae	*Erofurcata* (Villers, 1789)	introduced	32	25	10	6
Arachnida	Araneae	*Gibbaraneaoccidentalis* Wunderlich, 1989	endemic	2	264	289	280
Arachnida	Araneae	*Lasaeolaoceanica* Simon, 1883	endemic	0	3	0	0
Arachnida	Araneae	*Lathysdentichelis* (Simon, 1883)	native non-endemic	0	2	0	4
Arachnida	Araneae	*Leucognathaacoreensis* Wunderlich, 1992	endemic	4	21	27	19
Arachnida	Araneae	*Macaroeriscata* (Blackwall, 1867)	native non-endemic	0	24	11	15
Arachnida	Araneae	*Macaroerisdiligens* (Blackwall, 1867)	native non-endemic	0	6	5	11
Arachnida	Araneae	*Mangoraacalypha* (Walckenaer, 1802)	introduced	0	0	0	1
Arachnida	Araneae	*Metellinamerianae* (Scopoli, 1763)	introduced	0	7	0	0
Arachnida	Araneae	*Microlinyphiajohnsoni* (Blackwall, 1859)	native non-endemic	0	84	9	4
Arachnida	Araneae	*Osteariusmelanopygius* (O. Pickard-Cambridge, 1880)	introduced	0	1	0	0
Arachnida	Araneae	*Palliduphantesschmitzi* (Kulczynski, 1899)	native non-endemic	5	2	1	0
Arachnida	Araneae	*Pardosaacorensis* Simon, 1883	endemic	5	0	1	1
Arachnida	Araneae	*Pisauraacoreensis* Wunderlich, 1992	endemic	8	26	18	62
Arachnida	Araneae	*Porrhoclubionadecora* (Blackwall, 1859)	native non-endemic	0	17	2	5
Arachnida	Araneae	*Porrhoclubionagenevensis* (L. Koch, 1866)	introduced	1	16	0	1
Arachnida	Araneae	*Porrhommaborgesi* Wunderlich, 2008	endemic	2	0	2	0
Arachnida	Araneae	*Rugathodesacoreensis* Wunderlich, 1992	endemic	12	108	115	27
Arachnida	Araneae	*Savigniorrhipisacoreensis* Wunderlich, 1992	endemic	0	73	55	36
Arachnida	Araneae	*Segestriaflorentina* (Rossi, 1790)	introduced	0	1	0	0
Arachnida	Araneae	*Steatodanobilis* (Thorell, 1875)	native non-endemic	0	1	1	0
Arachnida	Araneae	*Tenuiphantesmiguelensis* (Wunderlich, 1992)	native non-endemic	304	18	1	1
Arachnida	Araneae	*Tenuiphantestenuis* (Blackwall, 1852)	introduced	27	45	0	1
Arachnida	Araneae	*Theridionmelanostictum* O. Pickard-Cambridge, 1876	introduced	0	1	0	0
Arachnida	Araneae	*Theridionmusivivum* Schmidt, 1956	native non-endemic	0	4	0	0
Arachnida	Araneae	*Walckenaeriagrandis* (Wunderlich, 1992)	endemic	2	13	0	0
Arachnida	Araneae	*Xysticuscor* Canestrini, 1873	native non-endemic	0	1	2	5
Arachnida	Opiliones	*Leiobunumblackwalli* Meade, 1861	native non-endemic	335	1412	279	174
Arachnida	Pseudoscorpiones	*Chthoniusischnocheles* (Hermann, 1804)	introduced	26	2	0	0
Arachnida	Pseudoscorpiones	*Ephippiochthoniustetrachelatus* (Preyssler, 1790)	introduced	0	1	0	0
Arachnida	Pseudoscorpiones	*Neobisiummaroccanum* Beier, 1930	introduced	0	8	0	0
Chilopoda	Geophilomorpha	*Geophilustruncorum* Bergsøe & Meinert, 1866	native non-endemic	1	0	0	0
Chilopoda	Geophilomorpha	*Strigamiacrassipes* (C.L. Koch, 1835)	native non-endemic	2	1	0	0
Chilopoda	Lithobiomorpha	*Lithobiuspilicornispilicornis* Newport, 1844	native non-endemic	91	1	0	0
Diplopoda	Julida	*Blaniulusguttulatus* (Fabricius, 1798)	introduced	176	0	0	0
Diplopoda	Julida	*Cylindroiuluspropinquus* (Porat, 1870)	introduced	10	0	0	0
Diplopoda	Julida	*Nopoiuluskochii* (Gervais, 1847)	introduced	11	1	0	0
Diplopoda	Julida	*Ommatoiulusmoreleti* (Lucas, 1860)	introduced	58	22	7	1
Diplopoda	Julida	*Proteroiulusfuscus* (Am Stein, 1857)	introduced	3	0	0	0
Diplopoda	Polydesmida	*Oxidusgracilis* (C.L. Koch, 1847)	introduced	3	0	0	0
Diplopoda	Polydesmida	*Polydesmuscoriaceus* Porat, 1870	introduced	13	0	0	0
Insecta	Archaeognatha	*Diltasaxicola* (Womersley, 1930)	native non-endemic	0	4	0	2
Insecta	Archaeognatha	*Trigoniophthalmusborgesi* Mendes, Gaju, Bach & Molero, 2000	endemic	1	160	21	105
Insecta	Blattodea	*Zethasimonyi* (Krauss, 1892)	native non-endemic	3	142	50	55
Insecta	Coleoptera	*Amischaanalis* (Gravenhorst, 1802)	indeterminate	0	2	0	0
Insecta	Coleoptera	*Anaspisproteus* Wollaston, 1854	native non-endemic	0	114	74	73
Insecta	Coleoptera	*Anisodactylusbinotatus* (Fabricius, 1787)	introduced	0	0	1	0
Insecta	Coleoptera	*Anobiumpunctatum* (De Geer, 1774)	introduced	0	1	0	0
Insecta	Coleoptera	*Anotylusnitidifrons* (Wollaston, 1871)	indeterminate	270	1	0	0
Insecta	Coleoptera	*Athetafungi* (Gravenhorst, 1806)	indeterminate	3	3	0	0
Insecta	Coleoptera	*Athetapasadenae* Bernhauer, 1906	indeterminate	0	1	0	0
Insecta	Coleoptera	*Athousazoricus* Platia & Gudenzi, 2002	endemic	3	12	0	0
Insecta	Coleoptera	*Brassicogethesaeneus* (Fabricius, 1775)	introduced	0	1	1	0
Insecta	Coleoptera	*Calacallessubcarinatus* (Israelson, 1984)	endemic	0	25	12	2
Insecta	Coleoptera	*Carpelimuscorticinus* (Gravenhorst, 1806)	indeterminate	6	8	0	0
Insecta	Coleoptera	*Carpelimustroglodytestroglodytes* (Erichson, 1840)	indeterminate	2	0	0	0
Insecta	Coleoptera	*Cartoderenodifer* (Westwood, 1839)	introduced	0	3	0	0
Insecta	Coleoptera	*Catopscoracinus* Kellner, 1846	native non-endemic	1	6	1	0
Insecta	Coleoptera	*Cedrorumazoricusazoricus* Borges & A.Serrano, 1993	endemic	27	0	0	0
Insecta	Coleoptera	*Cephenniumvalidum* Assing & Meybohm, 2021	native non-endemic	1	0	0	0
Insecta	Coleoptera	*Cercyonhaemorrhoidalis* (Fabricius, 1775)	introduced	1	0	0	0
Insecta	Coleoptera	*Coccinellaundecimpunctataundecimpunctata* Linnaeus, 1758	introduced	0	1	1	0
Insecta	Coleoptera	*Coccotrypescarpophagus* (Hornung, 1842)	introduced	0	1	1	0
Insecta	Coleoptera	*Creophilusmaxillosusmaxillosus* (Linnaeus, 1758)	indeterminate	0	0	1	0
Insecta	Coleoptera	*Cryptamorphadesjardinsii* (Guérin-Méneville, 1844)	introduced	0	1	0	1
Insecta	Coleoptera	*Drouetiusborgesiborgesi* (Machado, 2009)	endemic	1	67	3	1
Insecta	Coleoptera	*Dryopsalgiricus* (Lucas, 1846)	native non-endemic	1	1	1	1
Insecta	Coleoptera	*Epitrixhirtipennis* (Melsheimer, 1847)	introduced	0	1	0	0
Insecta	Coleoptera	*Gonipterusplatensis* (Marelli, 1926)	introduced	0	2	0	0
Insecta	Coleoptera	*Heteroderesazoricus* (Tarnier, 1860)	endemic	0	3	0	1
Insecta	Coleoptera	*Heteroderesvagus* Candèze, 1893	introduced	1	0	0	0
Insecta	Coleoptera	*Kalcapionsemivittatumsemivittatum* (Gyllenhal, 1833)	indeterminate	0	1	0	0
Insecta	Coleoptera	*Longitarsuskutscherai* (Rye, 1872)	introduced	0	3	0	0
Insecta	Coleoptera	*Mecinuspascuorum* (Gyllenhal, 1813)	introduced	0	1	2	0
Insecta	Coleoptera	*Notothectadryochares* (Israelson, 1985)	endemic	1	52	8	2
Insecta	Coleoptera	*Ocypusaethiops* (Waltl, 1835)	indeterminate	39	0	0	0
Insecta	Coleoptera	*Ocysharpaloides* (Audinet-Serville, 1821)	native non-endemic	0	0	5	0
Insecta	Coleoptera	*Paranchusalbipes* (Fabricius, 1796)	introduced	95	0	1	0
Insecta	Coleoptera	*Phloeonomuspunctipennis* Thomson, 1867	indeterminate	1	2	0	0
Insecta	Coleoptera	*Phyllotretastriolata* (Fabricius, 1803)	introduced	0	0	1	1
Insecta	Coleoptera	*Popilliajaponica* Newman, 1838	introduced	0	4	0	0
Insecta	Coleoptera	*Proteinusatomarius* Erichson, 1840	indeterminate	2	2	0	0
Insecta	Coleoptera	*Pseudoophonusrufipes* (De Geer, 1774)	introduced	1	0	0	0
Insecta	Coleoptera	*Pseudophloeophagustenaxborgesi* Stüben, 2022	endemic	2	74	27	15
Insecta	Coleoptera	*Psylliodesmarcida* (Illiger, 1807)	native non-endemic	0	1	0	0
Insecta	Coleoptera	*Sitonadiscoideus* Gyllenhal, 1834	introduced	0	2	0	0
Insecta	Coleoptera	*Sphenophorusabbreviatus* (Fabricius, 1787)	introduced	1	0	0	0
Insecta	Coleoptera	*Stelidotageminata* (Say, 1825)	introduced	47	0	1	0
Insecta	Coleoptera	*Stilbustestaceus* (Panzer, 1797)	native non-endemic	0	1	0	0
Insecta	Coleoptera	*Tachyporuschrysomelinus* (Linnaeus, 1758)	indeterminate	0	1	0	0
Insecta	Coleoptera	*Tachyporusnitidulus* (Fabricius, 1781)	indeterminate	1	0	0	0
Insecta	Coleoptera	*Tarphiusrelictus* Borges & Serrano, 2017	endemic	3	0	0	0
Insecta	Coleoptera	*Trechusterrabravensis* Borges, Serrano & Amorim, 2004	endemic	12	0	0	0
Insecta	Coleoptera	*Xyleborinusalni* Nijima, 1909	introduced	0	0	1	0
Insecta	Dermaptera	*Euborelliaannulipes* (Lucas, 1847)	introduced	0	6	0	0
Insecta	Dermaptera	*Forficulaauricularia* Linnaeus, 1758	introduced	1	3	0	0
Insecta	Ephemeroptera	*Cloeondipterum* (Linnaeus, 1761)	native non-endemic	0	0	1	0
Insecta	Hemiptera	*Acalyptaparvula* (Fallén, 1807)	native non-endemic	0	1	0	0
Insecta	Hemiptera	*Acizziauncatoides* (Ferris & Klyver, 1932)	introduced	0	512	82	62
Insecta	Hemiptera	*Anthocorisnemoralis* (Fabricius, 1794)	native non-endemic	0	1	0	0
Insecta	Hemiptera	*Aphrodeshamiltoni* Quartau & Borges, 2003	endemic	22	5	0	0
Insecta	Hemiptera	*Buchananiellacontinua* (White, 1880)	introduced	0	1	0	0
Insecta	Hemiptera	*Campyloneuravirgula* (Herrich-Schaeffer, 1835)	native non-endemic	1	37	35	17
Insecta	Hemiptera	*Cinarajuniperi* (De Geer, 1773)	native non-endemic	0	90	3	7
Insecta	Hemiptera	*Cixiusazoterceirae* Remane & Asche, 1979	endemic	6	1926	915	1304
Insecta	Hemiptera	*Cyphopterumadscendens* (Herrich-Schäffer, 1835)	native non-endemic	0	140	73	23
Insecta	Hemiptera	*Eupteryxazorica* Ribaut, 1941	endemic	0	2	0	2
Insecta	Hemiptera	*Eupteryxfilicum* (Newman, 1853)	native non-endemic	0	20	4	0
Insecta	Hemiptera	*Fulviusborgesi* Chérot, Ribes & Gorczyca, 2006	introduced	0	0	0	1
Insecta	Hemiptera	*Heterotomaplanicornis* (Pallas, 1772)	native non-endemic	0	1	0	0
Insecta	Hemiptera	*Kelisiaribauti* Wagner, 1938	native non-endemic	0	10	4	3
Insecta	Hemiptera	*Kleidocerysericae* (Horváth, 1909)	native non-endemic	0	329	13	10
Insecta	Hemiptera	*Loriculacoleoptrata* (Fallén, 1807)	native non-endemic	0	4	4	2
Insecta	Hemiptera	*Megamelodesquadrimaculatus* (Signoret, 1865)	native non-endemic	43	0	0	1
Insecta	Hemiptera	*Monalocorisfilicis* (Linnaeus, 1758)	native non-endemic	0	112	15	10
Insecta	Hemiptera	*Nabispseudoferusibericus* Remane, 1962	native non-endemic	0	9	4	4
Insecta	Hemiptera	*Oriuslaevigatuslaevigatus* (Fieber, 1860)	native non-endemic	0	1	0	0
Insecta	Hemiptera	*Pilophorusperplexus* Douglas & Scott, 1875	native non-endemic	0	0	42	0
Insecta	Hemiptera	*Pinalitusoromii* J. Ribes, 1992	endemic	0	34	29	48
Insecta	Hemiptera	*Rhopalosiphoninuslatysiphon* (Davidson, 1912)	introduced	10	0	0	0
Insecta	Hemiptera	*Saldulapalustris* (Douglas, 1874)	native non-endemic	0	0	1	1
Insecta	Hemiptera	*Scolopostethusdecoratus* (Hahn, 1833)	native non-endemic	0	0	1	0
Insecta	Hemiptera	*Siphantaacuta* (Walker, 1851)	introduced	0	85	7	8
Insecta	Hemiptera	*Strophingiaharteni* Hodkinson, 1981	endemic	0	23	6	11
Insecta	Hemiptera	*Triozalaurisilvae* Hodkinson, 1990	native non-endemic	1	329	376	821
Insecta	Hymenoptera	*Hypoponeraeduardi* (Forel, 1894)	native non-endemic	0	0	1	2
Insecta	Hymenoptera	*Lasiusgrandis* Forel, 1909	native non-endemic	52	171	13	20
Insecta	Hymenoptera	*Monomoriumcarbonarium* (Smith, 1858)	native non-endemic	0	0	0	3
Insecta	Hymenoptera	*Tetramoriumcaespitum* (Linnaeus, 1758)	native non-endemic	0	4	0	0
Insecta	Lepidoptera	*Argyresthiaatlanticella* Rebel, 1940	endemic	4	0	0	0
Insecta	Lepidoptera	*Ascotisfortunataazorica* Pinker, 1971	endemic	1	0	0	0
Insecta	Lepidoptera	*Mythimnaunipuncta* (Haworth, 1809)	native non-endemic	1	0	0	0
Insecta	Neuroptera	*Hemerobiusazoricus* Tjeder, 1948	endemic	0	64	49	34
Insecta	Phasmida	*Carausiusmorosus* (Sinéty, 1901)	introduced	0	3	0	0
Insecta	Psocodea	*Atlantopsocusadustus* (Hagen, 1865)	native non-endemic	0	14	11	6
Insecta	Psocodea	*Bertkauialucifuga* (Rambur, 1842)	native non-endemic	0	16	4	2
Insecta	Psocodea	*Ectopsocusbriggsi* McLachlan, 1899	introduced	2	262	71	104
Insecta	Psocodea	*Ectopsocusstrauchi* Enderlein, 1906	native non-endemic	0	1	1	0
Insecta	Psocodea	*Elipsocusazoricus* Meinander, 1975	endemic	0	113	68	40
Insecta	Psocodea	*Elipsocusbrincki* Badonnel, 1963	endemic	0	56	37	146
Insecta	Psocodea	*Lachesillagreeni* (Pearman, 1933)	introduced	0	0	0	2
Insecta	Psocodea	*Trichopsocusclarus* (Banks, 1908)	native non-endemic	2	307	90	33
Insecta	Psocodea	*Valenzuelaburmeisteri* (Brauer, 1876)	native non-endemic	0	47	17	5
Insecta	Psocodea	*Valenzuelaflavidus* (Stephens, 1836)	native non-endemic	2	1089	266	214
Insecta	Strepsiptera	*Elenchustenuicornis* (Kirby, 1815)	native non-endemic	0	0	0	1
Insecta	Thysanoptera	*Anisopilothripsvenustulus* (Priesner, 1923)	introduced	0	1	0	0
Insecta	Thysanoptera	*Ceratothripsericae* (Haliday, 1836)	native non-endemic	0	61	13	7
Insecta	Thysanoptera	*Heliothripshaemorrhoidalis* (Bouché, 1833)	introduced	0	13	2	1
Insecta	Thysanoptera	*Hercinothripsbicinctus* (Bagnall, 1919)	introduced	0	1	0	0
Insecta	Thysanoptera	*Hoplothripscorticis* (De Geer, 1773)	native non-endemic	0	65	49	27
Insecta	Trichoptera	*Limnephilusatlanticus* Nybom, 1948	endemic	7	3	5	0

**Table 3.** Pairwise comparisons of adult arthropods abundance between exotic forest and native forest across different strata (EPI: epigean layer, GRD: ground layer, UND: understorey layer, CAN: canopy layer) using Wilcoxon rank sum tests. The table presents the sample sizes (n1 and n2), test statistic values and significance levels (*p < 0.05, **p < 0.01, ns = not significant) as well as the effect size and magnitude, based on 1000 replications for the significant comparisons.

Strata	Forest type 1	Forest type 2	n1	n2	W	Significance	Effect size	Magnitude
EPI	Exotic forest	Native forest	10	10	62.5	ns	---	---
GRD	Exotic forest	Native forest	10	10	84	**	0.57	large
UND	Exotic forest	Native forest	10	8	15	*	0.52	large
CAN	Exotic forest	Native forest	10	10	9	**	0.69	large

**Table 4.** Statistical comparison of arthropod order abundance between exotic and native forests across different forest strata (EPI: epigean layer, GRD: ground layer, UND: understorey layer, CAN: canopy layer). n1 and n2 represent sample sizes for exotic and native forests, respectively. p-values are derived from statistical tests (Wilcoxon rank sum tests), with significance levels indicated as: ns (not significant), * (p < 0.05), ** (p < 0.01), *** (p < 0.001).

Order	Strata	n1	n2	W	p-value	Significance
Araneae	EPI	10	10	31	0.162	ns
Araneae	GRD	10	10	19	0.0185	*
Araneae	UND	10	8	2	0.00081	***
Araneae	CAN	10	10	35	0.272	ns
Archaeognatha	EPI	10	10	45	0.368	ns
Archaeognatha	GRD	10	10	31	0.101	ns
Archaeognatha	UND	10	8	25	0.0474	*
Archaeognatha	CAN	10	10	40	0.399	ns
Blattodea	GRD	10	10	42.5	0.572	ns
Blattodea	UND	10	8	20	0.0174	*
Blattodea	CAN	10	10	46	0.67	ns
Coleoptera	EPI	10	10	81	0.0211	*
Coleoptera	GRD	10	10	41	0.529	ns
Coleoptera	UND	10	8	68	0.0117	*
Coleoptera	CAN	10	10	100	0.000178	***
Hemiptera	EPI	10	10	5.5	0.00038	***
Hemiptera	GRD	10	10	59	0.529	ns
Hemiptera	UND	10	8	27	0.274	ns
Hemiptera	CAN	10	10	13	0.00578	**
Hymenoptera	EPI	10	10	80	0.00597	**
Hymenoptera	GRD	10	10	59	0.517	ns
Hymenoptera	UND	10	8	39	0.962	ns
Hymenoptera	CAN	10	10	73	0.0626	ns
Julida	EPI	10	10	52	0.901	ns
Julida	GRD	10	10	55	0.69	ns
Julida	UND	10	8	35	0.314	ns
Lithobiomorpha	EPI	10	10	14.5	0.00789	**
Lithobiomorpha	GRD	10	10	45	0.368	ns
Neuroptera	GRD	10	10	51	0.968	ns
Neuroptera	UND	10	8	57	0.138	ns
Neuroptera	CAN	10	10	82	0.0164	*
Opiliones	EPI	10	10	60.5	0.394	ns
Opiliones	GRD	10	10	75	0.062	ns
Opiliones	UND	10	8	37	0.823	ns
Opiliones	CAN	10	10	66.5	0.22	ns
Psocodea	EPI	10	10	65	0.0779	ns
Psocodea	GRD	10	10	54	0.796	ns
Psocodea	UND	10	8	60	0.0831	ns
Psocodea	CAN	10	10	77	0.0433	*
Thysanoptera	GRD	10	10	66	0.238	ns
Thysanoptera	UND	10	8	63.5	0.0325	*
Thysanoptera	CAN	10	10	47	0.843	ns
